# Epidural Spinal Cord Stimulation Acutely Modulates Lower Urinary Tract and Bowel Function Following Spinal Cord Injury: A Case Report

**DOI:** 10.3389/fphys.2018.01816

**Published:** 2018-12-18

**Authors:** Matthias Walter, Amanda H. X. Lee, Alex Kavanagh, Aaron A. Phillips, Andrei V. Krassioukov

**Affiliations:** ^1^International Collaboration on Repair Discoveries, Faculty of Medicine, The University of British Columbia, Vancouver, BC, Canada; ^2^Department of Urologic Sciences, The University of British Columbia, Vancouver, BC, Canada; ^3^Department of Physiology and Pharmacology, Libin Cardiovascular Institute of Alberta, Hotchkiss Brain Institute, University of Calgary, Calgary, AB, Canada; ^4^Department of Cardiac Sciences, Libin Cardiovascular Institute of Alberta, Hotchkiss Brain Institute, University of Calgary, Calgary, AB, Canada; ^5^Department of Clinical Neurosciences, Libin Cardiovascular Institute of Alberta, Hotchkiss Brain Institute, University of Calgary, Calgary, AB, Canada; ^6^Division of Physical Medicine and Rehabilitation, Faculty of Medicine, The University of British Columbia, Vancouver, BC, Canada; ^7^G.F. Strong Rehabilitation Centre, Vancouver, BC, Canada

**Keywords:** epidural spinal cord stimulation, neurogenic bowel dysfunction, neurogenic lower urinary tract dysfunction, spinal cord injury, urodynamic investigation

## Abstract

Regaining control of autonomic functions such as those of the cardiovascular system, lower urinary tract and bowel, rank among the most important health priorities for individuals living with spinal cord injury (SCI). Recently our research provided evidence that epidural spinal cord stimulation (ESCS) could acutely modulate autonomic circuits responsible for cardiovascular function after SCI. This finding raised the question of whether ESCS can be used to modulate autonomic circuits involved in lower urinary tract and bowel control after SCI. We present the case of a 32-year-old man with a chronic motor-complete SCI (American Spinal injury Association Impairment Scale B) at the 5^th^ cervical spinal segment. He sustained his injury during a diving accident in 2012. He was suffering from neurogenic lower urinary tract and bowel dysfunction. Epidural stimulation of the lumbosacral spinal cord immediately modulated both functions without negatively affecting the cardiovascular system. Specifically, the individual’s bowel function was assessed using different pre-set configurations and stimulation parameters in a randomized order. Compared to the individual’s conventional bowel management approach, ESCS significantly reduced the time needed for bowel management (*p* = 0.039). Furthermore, depending on electrode configuration and stimulation parameters (i.e., amplitude, frequency, and pulse width), ESCS modulated detrusor pressure and external anal sphincter/pelvic floor muscle tone to various degrees during urodynamic investigation. Although, ESCS is currently being explored primarily for restoring ambulation, our data suggest that application of this neuroprosthetic intervention may provide benefit to lower urinary tract and bowel function in individuals with SCI. To fully capitalize on the potential of improving lower urinary tract and bowel function, further research is needed to better understand the neuronal pathways and identify optimal stimulation configurations and parameters.

## Introduction

When individuals living with spinal cord injury (SCI) rank their health priorities, regaining autonomic functions such as those of the lower urinary tract and bowel, are consistently considered more important than walking again (Anderson, 2004). Lower urinary tract and bowel dysfunction following SCI results from the partial or total loss of supraspinal control (Fowler et al., 2008). Lower urinary tract dysfunction frequently occurs (Schops et al., 2015) and is often characterized by urinary incontinence as a result from spontaneous uninhibited contractions of the detrusor, known as neurogenic detrusor overactivity (Panicker et al., 2015). The latter poses a significant health risk to patients with SCI by repeatedly increasing intravesical pressures, which can result in morphological changes of the entire urinary tract and long-term increased risk of upper urinary tract complications (Hackler, 1977; Panicker et al., 2015). The latter includes vesico-uretero-renal reflux, hydronephrosis and impairment of renal functions or even terminal renal failure (Hackler, 1977; Panicker et al., 2015). Furthermore, the inability to voluntarily empty the bladder is often impaired in this population (Fowler et al., 2008). Bowel dysfunction following SCI is typically displayed by reduced gut/intestinal motility leading to a much longer transit time (Krassioukov et al., 2010). Therefore, it comes as no surprise that the average time needed for bowel management in this population ranges from 1.5 h (Stiens et al., 1998; Ayas et al., 2006) to 2.4 h (Frisbie, 1997). Furthermore, the majority of individuals following SCI rely on additional measures such as suppositories, laxatives, and/or digital stimulation to facilitate bowel evacuation (Krassioukov et al., 2010). Improving these functions will not only improve quality of life (Patel et al., 2016), but also will reduce the duration of care (bowel routines are often more than 1 h) (Rosito et al., 2002) and the prevalence of artificial bladder emptying strategies including intermittent or indwelling catheterization (Cameron et al., 2010; Jeong et al., 2010). The latter is associated with high risk of urinary tract infection (Linsenmeyer, 2018) and bladder cancer (Welk et al., 2013), and is a considerable economic health care burden (White et al., 2017; Welk et al., 2018).

## Background

Our research team provided evidence that stimulation approaches including epidural (West et al., 2018) or transcutaneous (Phillips et al., 2018) could specifically modulate autonomic pathways responsible for cardiovascular control in individuals with motor-complete SCI. These findings have recently been replicated using epidural stimulation (Aslan et al., 2018). In addition, recent rodent data indicated that epidural stimulation may be capable of modulating spinal cord circuits responsible for lower urinary tract and bowel control after SCI (Gad et al., 2016). Given these promising findings, we hypothesized that direct spinal cord stimulation could also affect spinal cord circuits controlling lower urinary tract and bowel function. While one case series recently reported on improved voiding function (Herrity et al., 2018), the effects of epidural spinal cord stimulation (ESCS) on bowel function in individuals following SCI have not been explored in humans. Here, we report that ESCS of the lumbosacral spinal cord can acutely modulate lower urinary tract and bowel function in an individual with motor-complete SCI.

## Case Presentation

### Participant

The participant was a 32-year-old man with a well-documented history of autonomic dysfunction (Krassioukov et al., 2012) including autonomic dysreflexia as well as neurogenic lower urinary tract and bowel dysfunction as a consequence of his motor-complete, sensory incomplete SCI (C5, American Spinal Injury Association Impairment Scale B) (Kirshblum et al., 2011) sustained in a diving accident in 2012. The participant was relying on intermittent catheterization to empty his bladder and suppository use as well as digital stimulation to facilitate bowel routine.

### Neurostimulator

**FIGURE 1:**
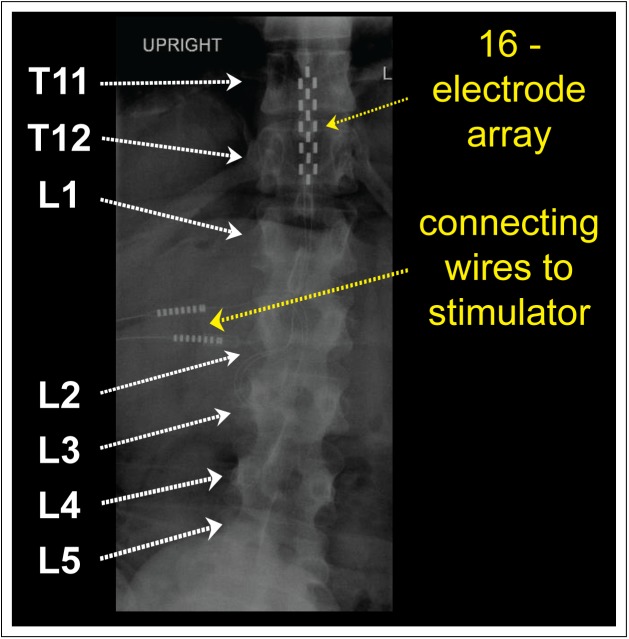
Anatomical placement of 16-electrode array: conventional radiography of the thoracic/lumbar spine displays the position of 16-electrode array (vertebral levels T11–L1).

With the intent to improve his motor function, the participant received an ESCS unit and 16-electrode array (RestoreAdvanced SureScan MRI neurostimulator, Specify 5-6-5, Medtronic, Minneapolis, MN, United States) in 2016. The neurostimulator was equipped with numerous pre-set stimulation programs comprising different electrode configurations and stimulation parameters (i.e., frequency, pulse width and intensity). Compared to no stimulation (Panel A), we applied a variety of pre-set stimulation programs (Panels B to H) during our assessments. Each stimulation program was designed to activate specific groups of skeletal muscles responsible for: (Panel B) left ankle dorsiflexion and left hip/knee flexion, (Panel C) left hip/knee flexion, (Panel D) left knee extension, (Panel E) right knee extension, (Panel F) right step forward, (Panel G) right ankle dorsiflexion and right hip/knee flexion, and (Panel H) bilateral trunk muscle activation. The participant utilizes the stimulator as needed by simply turning it on and selecting a program. In contrast to frequency and pulse width, which were pre-set, the participant can change the intensity of each program manually as needed. Prior to our investigation, a radiologist confirmed correct placement of the 16-electrode array at vertebral levels T11 to L1 via conventional radiography (Figure 1).

### Assessment of Lower Urinary Tract Function

Urodynamic investigation along with surface external anal sphincter/pelvic floor electromyography is the gold standard to assess lower urinary tract function (Groen et al., 2016). First, baseline urodynamic investigation (Aquarius TT, Laborie Model 94-R03-BT, Montreal, QC, Canada) was performed in accordance with the International Continence Society’s ‘Good Urodynamic Practices’ (Schafer et al., 2002) to evaluate the current extent of neurogenic lower urinary tract dysfunction. Since the participant is not able to void spontaneously, we only conducted an experimental urodynamic investigation to evaluate if ESCS of the lumbosacral spinal cord exerts an acute effect on detrusor pressure, as well as external anal sphincter and pelvic floor muscle tone during the storage phase. For this, we prefilled the bladder to a volume of 250 mL (i.e., 60% of maximum cystometric capacity from the first urodynamic investigation), without eliciting neurogenic detrusor overactivity or autonomic dysreflexia. Then, pre-set stimulation programs (B to F) were applied for at least 90 s each.

### Cardiovascular Monitoring

Concurrent to the urodynamic investigation, we continuously recorded beat-by-beat blood pressure, via finger photoplethysmography (Finometer PRO, Finapres Medical Systems, Amsterdam, Netherlands) corrected to brachial pressure (CARESCAPE V100, GE Healthcare, Milwaukee, WI, United States), and one-lead electrocardiogram (eML 132; ADInstruments, Colorado Springs, CO, United States) for heart rate in order to detect autonomic dysreflexia (Walter et al., 2018).

Autonomic dysreflexia is defined as a response to noxious or non-noxious stimuli below the level of SCI that typically occurs primarily in people with an SCI at or above the T6 spinal segment. This condition is characterized by an increase in systolic blood pressure of 20 mmHg or more above baseline (Krassioukov et al., 2012). Autonomic dysreflexia is highly prevalent in this population (Curt et al., 1997) and can occur more than 40 times per day (Hubli et al., 2015). As blood pressure can rise above 300 mmHg, autonomic dysreflexia is a potentially life-threatening condition that can result in stroke, seizure, myocardial ischemia, or even death (Wan and Krassioukov, 2014). Prior to the urodynamic investigation, baseline measurement of brachial blood pressure and heart rate was performed three times within 5 min and averaged. All lower urinary tract assessments were performed in the supine position.

### Assessment of Bowel Function

To assess the magnitude of bowel dysfunction in individuals following SCI, ‘*The Neurogenic Bowel Dysfunction (NBD) Score*’ questionnaire has been shown to provide a clinically meaningful outcome measure with a good reproducibility and validity (Krogh et al., 2006).

This standardized questionnaire comprises 10 questions focusing on defecation (i.e., frequency, duration, and clinical symptoms), constipation (i.e., use of aiding medication and digital stimulation), fecal incontinence (i.e., frequency, aiding medication, and flatus) and peri-anal skin problems. The consequential NBD score relates to four different neurogenic bowel dysfunction severity levels (i.e., score 0–6 = very minor, 7–9 = minor, 10–13 = moderate, and 14–47 = severe). In addition to the NBD score, the questionnaire assesses the patient’s general satisfaction regarding current bowel function through one item (i.e., a numeric rating scale: from 0 = total dissatisfaction to 10 = total satisfaction). We next objectively tested various stimulation programs compared to conventional bowel routine (i.e., suppository use only) in terms of time required for bowel management. In a randomized order, three different stimulation programs (Panels E, G, and H) representing different electrode configurations and stimulation parameters as well as conventional bowel routine (Panel A) were each assessed three times (i.e., overall 12 trials) within a period of 1 month. To assess and compare the time required for bowel management, the participant was instructed to record the time from *‘suppository insertion’* to *‘when bowel evacuation was completed’* at his home. The neurostimulator was turned on after the suppository insertion and turned off following completion of bowel evacuation.

### Epidural Spinal Cord Stimulation Acutely Modulates Lower Urinary Tract and Bowel Function

**FIGURE 2:**
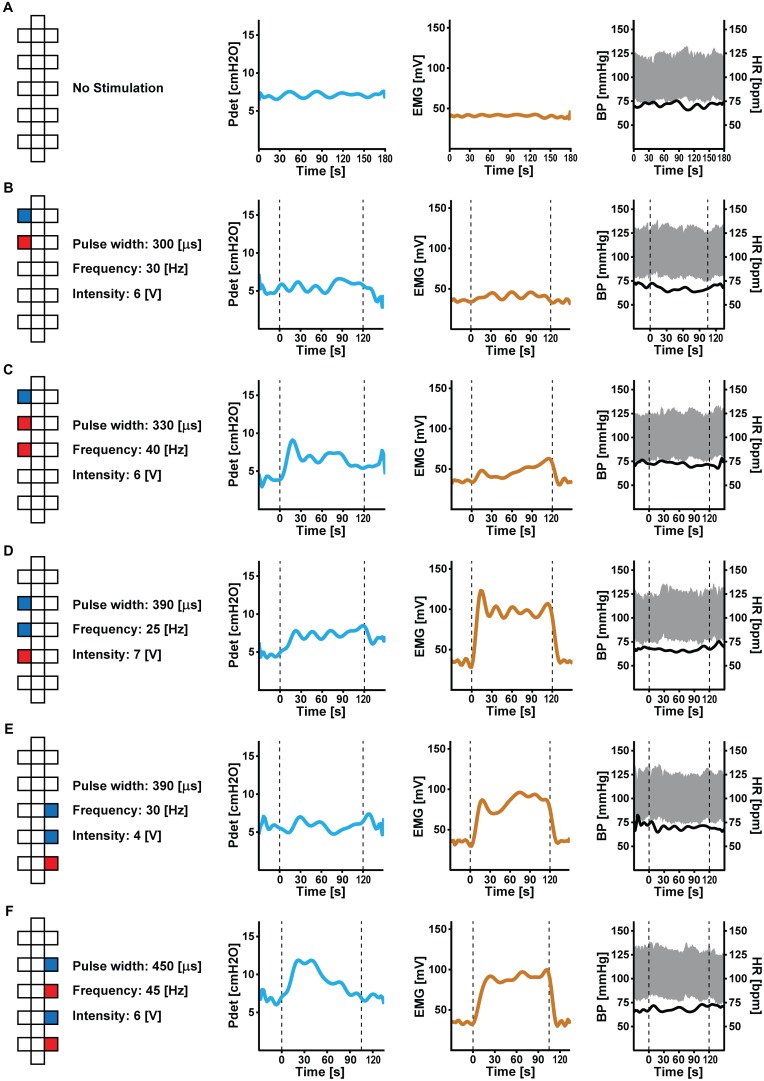
Effect of acute epidural spinal cord stimulation (ESCS) on lower urinary tract function: at baseline **(A)**, detrusor pressure (Pdet, in blue), external anal sphincter/pelvic floor muscle tone (EMG, in brown) and cardiovascular parameters [blood pressure (BP, in gray) and heart rate (HR, in black)] are stable. Depending on pre-set parameters (i.e., electrode configuration, amplitude, frequency, and pulse width), ESCS **(B–F)** increased external anal sphincter/pelvic floor muscle tone and detrusor pressure to various degrees. Cardiovascular responses remained stable (i.e., without and during ESCS). Dashed lines indicate start and stop of ESCS. Electrode configuration are as follows: red = cathode, blue = anode, and white = inactive.

**FIGURE 3:**
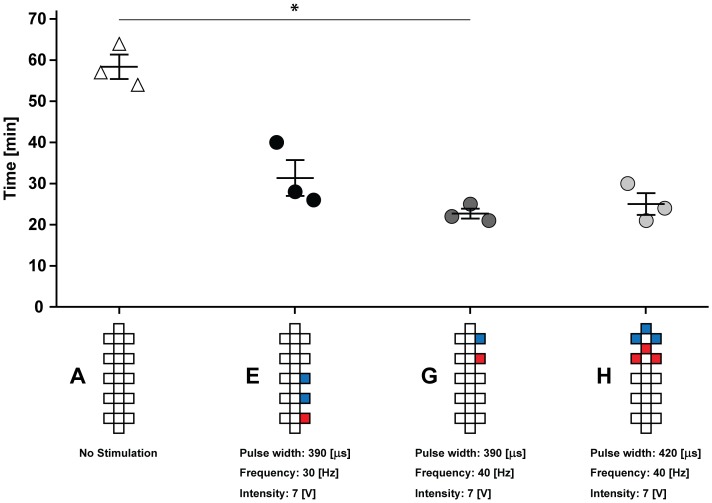
Effect of acute ESCS on bowel function: compared to conventional bowel routine (A), Kruskal–Wallis one-way analysis of variance revealed a significant (*p* = 0.039) reduction in the time required for bowel management when applying ESCS. ^∗^*Post hoc* analysis (Dunn test for multiple comparison using Bonferroni correction) confirmed significant effect of stimulation (G) compared to conventional bowel routine (23 ± 1 vs. 58 ± 3 min, *p* = 0.046). The other two stimulation programs (E,H) also reduced time required for bowel management (i.e., 25 ± 3 and 31 ± 4 min), but did not yield statistical significance (*p* > 0.05) compared to conventional bowel routine. Time needed for bowel management is presented as mean and standard error. Electrode configuration are as follows: red = cathode, blue = anode, and white = inactive.

Depending on electrode configuration and stimulation parameters (i.e., amplitude, frequency, and pulse width), ESCS modulated lower urinary tract (Figure 2) and bowel function (Figure 3) to various degrees.

Epidural spinal cord stimulation applied to caudal parts of the array increased external anal sphincter/pelvic floor muscle tone and detrusor pressure, while configurations stimulating the rostral part of the array had more modest or no effects compared to baseline. Blood pressure and heart rate remained stable during ESCS and autonomic dysreflexia did not occur.

Compared to conventional bowel routine (i.e., suppository alone), ESCS (plus suppository) significantly expedited bowel management (*p* = 0.039). On average, ESCS reduced the time required for bowel routine by more than 55% (i.e., 26 vs. 58 min). Furthermore, ESCS decreased the severity of neurogenic bowel dysfunction from severe to minor as evidenced by a reduction in NDB Score from 15 to 8, as well as improved the general satisfaction scale from 5 to 8.

## Discussion

Our data indicate that acute lumbosacral ESCS activates autonomic and motor spinal cord circuits that affect the lower urinary tract, external, anal sphincter/pelvic floor, and bowel function in individuals after chronic motor-complete SCI.

Following SCI, the majority of individuals experience partial or complete loss of their supraspinal bowel and bladder control. These conditions are known as neurogenic lower urinary tract and bowel dysfunction. The latter commonly leads to a longer transit time. Furthermore, individuals with SCI are often dependent on additional strategies to facilitate bowel evacuation (Krassioukov et al., 2010).

As in the case of our participant, before implantation he experienced a very lengthy bowel routine requiring suppositories and digital stimulation. After implantation, when stimulation was not applied, his bowel routine duration remains lengthy and still requires additional measures. Here, we observed with acute epidural stimulation (plus suppository) that time needed for bowel routine was reduced by more than 55% compared to conventional bowel routine (i.e., suppository alone). In order to assess the time required for bowel management (i.e., stimulation versus no stimulation), we standardized the start of assessment as the time point of ‘*suppository insertion.’* Given this, these findings do not permit a discussion on the effect of ESCS alone. Future work should investigate whether ESCS is capable of reducing the reliance on additional measures such as suppositories, laxatives, and/or digital stimulation to facilitate bowel evacuation. Although this study was not geared toward determining the underlying mechanisms, our current understanding of bowel function indicates the improvement is due to contraction of abdominal muscles, which increases intra-abdominal pressure and promotes bowel evacuation (Korsten et al., 2004). Further mechanistic research is crucial to truly understand these effects on bowel function. To our knowledge, a significant improvement in bowel function and its related quality of life due to acute ESCS has not been previously reported. The pre-set programs utilized to investigate the effects of ESCS on bowel function are well within the tolerated exposure (i.e., durations and intensity of stimulation), suggesting this strategy has the potential to become a viable therapeutic option.

Lack of lower urinary tract control following SCI frequently results in urinary incontinence and an inability to voluntarily empty the bladder (Groen et al., 2016; Wyndaele, 2016). In the present case, ESCS, with the sole intention of activating skeletal muscles, was capable of modulating lower urinary tract function. Although we investigated the effect of ESCS on lower urinary function at a single time point, these data clearly demonstrate that external anal sphincter and pelvic floor muscle tone, as well as detrusor pressure, were modulated by multiple stimulation programs. Importantly, blood pressure and heart rate were stable in response to ESCS and did not exceed the threshold for autonomic dysreflexia.

Herrity et al. (2018) tested different electrode configurations and stimulation parameters (frequency and pulse width) to optimize voiding efficiency following SCI. Whether epidural stimulation can be optimized for further enhanced function is outside of the scope of this case report, as we were testing the potential for pre-set stimulation programs, designed to activate specific groups of skeletal muscles, to modulate lower urinary tract and bowel function. Studies focused on volitional movement and cardiovascular control have shown that ESCS activates dorsal afferents (Capogrosso et al., 2013), which is thought to increase the central excitability below the level of injury and awaken spared dormant pathways that convey supraspinal input to the spinal circuits (Courtine et al., 2009; Capogrosso et al., 2013; Angeli et al., 2014). The present observations are congruent with these aforementioned mechanisms, potentially explaining improved lower urinary tract and bowel function after SCI. Others groups (Gad et al., 2018; Niu et al., 2018) have observed the beneficial effects of non-invasive spinal cord stimulation on lower urinary tract function in individuals following SCI, akin to those demonstrated with epidural stimulation (Herrity et al., 2018).

Despite these emerging findings, further research is necessary to reveal how autonomic connections are altered after injury, and to fully identify the underlying mechanistic pathways responsible for observed functional autonomic improvements with spinal cord stimulation. This will allow us to find the optimal stimulation parameters and target the appropriate structures to improve these crucial functions in those living with SCI.

## Availability of Data and Materials

The datasets used and/or analyzed during the current study are available from the corresponding author on request.

## Ethics Statement

The University of British Columbia Clinical Research Ethics Board approved this study. The participant provided his written informed consent according to the Helsinki II declaration including research dissemination, such as publication.

## Author Contributions

According to the guidelines of the International Committee of Medical Journal Editors (ICMJE), all authors contributed to the four criteria. MW, AAP, AHXL, AK, and AVK study concept and design, acquisition of data, and analysis and interpretation of data. MW, AAP, and AVK statistical analysis and drafting of the manuscript. AHXL and AK critical revision of the manuscript for important intellectual content.

## Conflict of Interest Statement

The authors declare that the research was conducted in the absence of any commercial or financial relationships that could be construed as a potential conflict of interest.
